# An end-to-end heterogeneous graph attention network for *Mycobacterium tuberculosis* drug-resistance prediction

**DOI:** 10.1093/bib/bbab299

**Published:** 2021-08-20

**Authors:** Yang Yang, Timothy M Walker, Samaneh Kouchaki, Chenyang Wang, Timothy E A Peto, Derrick W Crook, CRYPTIC Consortium, David A Clifton

**Affiliations:** Institute of Biomedical Engineering, Department of Engineering Science, University of Oxford, Oxford, OX3 7DQ, UK; Oxford-Suzhou Centre for Advanced Research, Suzhou, 215123, China; Oxford University Clinical Research Unit, Ho Chi Minh City, Vietnam; Centre for vision, Speech, and Signal processing, University of Surrey, Guildford, UK; Institute of Biomedical Engineering, Department of Engineering Science, University of Oxford, Oxford, OX3 7DQ, UK; Nuffield Department of Medicine, University of Oxford, John Radcliffe Hospital Headley Way, OX3 9DU, Oxford, UK; Nuffield Department of Medicine, University of Oxford, John Radcliffe Hospital Headley Way, OX3 9DU, Oxford, UK; NIHR Oxford Biomedical Research Centre, John Radcliffe Hospital, Headley Way Headington, OX3 9DU, Oxford, UK; Institute of Biomedical Engineering, Department of Engineering Science, University of Oxford, Oxford, OX3 7DQ, UK; Oxford-Suzhou Centre for Advanced Research, Suzhou, 215123, China

**Keywords:** graph learning, deep learning, antibiotic resistance, machine learning, hierarchical attention, genetic data analysis

## Abstract

Antimicrobial resistance (AMR) poses a threat to global public health. To mitigate the impacts of AMR, it is important to identify the molecular mechanisms of AMR and thereby determine optimal therapy as early as possible. Conventional machine learning-based drug-resistance analyses assume genetic variations to be homogeneous, thus not distinguishing between coding and intergenic sequences. In this study, we represent genetic data from *Mycobacterium tuberculosis* as a graph, and then adopt a deep graph learning method—heterogeneous graph attention network (‘HGAT–AMR’)—to predict anti-tuberculosis (TB) drug resistance. The HGAT–AMR model is able to accommodate incomplete phenotypic profiles, as well as provide ‘attention scores’ of genes and single nucleotide polymorphisms (SNPs) both at a population level and for individual samples. These scores encode the inputs, which the model is ‘paying attention to’ in making its drug resistance predictions. The results show that the proposed model generated the best area under the receiver operating characteristic (AUROC) for isoniazid and rifampicin (98.53 and 99.10%), the best sensitivity for three first-line drugs (94.91% for isoniazid, 96.60% for ethambutol and 90.63% for pyrazinamide), and maintained performance when the data were associated with incomplete phenotypes (i.e. for those isolates for which phenotypic data for some drugs were missing). We also demonstrate that the model successfully identifies genes and SNPs associated with drug resistance, mitigating the impact of resistance profile while considering particular drug resistance, which is consistent with domain knowledge.

## 1 Introduction

Antimicrobial resistance (AMR) is a global public health challenge that is impacting the way infections are treated. Mycobacterium tuberculosis (MTB), which kills more people each year than any other single pathogen, is not an exception. Timely prediction of resistance or susceptibility of a bacterial sample (or ‘isolate’) to anti-TB drugs is key to personalised treatment. As bacterial AMR is usually genetically encoded. There are two major ways to make a fast prediction of AMR for previously unseen isolates using genetic data [1]: (i) making predictions on the presence or absence of a defined catalogue of mutations or (ii) developing computational models to map the genomic data to phenotypes (e.g. resistance / susceptibility to drugs), and let the output of the model determine the prediction. The former is the route taken to date in clinical practice and depends entirely on domain knowledge. While catalogues perform well for well-studied drugs, they are necessarily less complete for less well-studied drugs. Machine learning (ML) has been extensively investigated in the study of AMR to predict resistance phenotypes directly from genotypes [2]. This has typically involved classic models [3] such as support vector machines, logistic regression, random forests and others, as well as deep learning models [4–6] (incorporating stacks of layers of neural networks). These existing ML models focus on two issues: (i) improving prediction performance and (ii) identifying the predictive genetic variables [7]. Conventional ML-based drug-resistance models are trained on information about the presence or absence of genetic variables, which assume genetic variations to be homogeneous and do not differentiate between coding and intergenic sequences. They also do not take into account that different genetic mutations in the same gene are involved in coding for the same protein, or that some genes contribute more than others to resistance to a particular drug. The existing ML methods thus often identify the most predictive variables (via association) instead of the most interpretable ones. The most predictive mutations could be those that are unlikely to be biologically related to a particular drug. For example, we frequently see that resistance-associated single nucleotide polymorphisms (SNPs) for one drug are ranked as being important for resistance to another drug because patterns of co-occurring resistance to drugs can emerge for epidemiological reasons. A classic example if this would be the fact that rifampicin (RIF) resistance rarely occurs in the absence of isoniazid (INH) resistance, even though the mechanisms of resistance are unrelated. Consequently, predictive mutations of the former also predict the latter yet in no way contribute to the resistance. In practice, it is common that not all samples are tested phenotypically for resistance to all drugs [8]. Samples are often tested to first-line drugs and then only to second-line drugs if resistance to the former is detected. Existing models typically involve the removal of samples with missing phenotypes from analyses, which substantially reduces the number of examples available for multi-drug resistance analysis and reduces the statistical power of the resulting conclusions. In this paper, we propose a graph learning model based on heterogeneous graph attention network (HGAT) [9] to model the full available dataset with improved interpretability—yielding better understanding of the link between drug resistance and factors with which it is associated. (We note in passing that such methods do not claim causality, which requires different approaches beyond the scope of this paper.) This is the first study to format genetic samples as a heterogeneous graph at the level of SNP granularity. In comparison with existing ML models for predicting single-drug resistance and antibiotic profiles, the proposed methods show better prediction performance. Furthermore, the HGAT model allows us to quantify multi-scale contributions of genetic variables from gene and mutation levels for either individual samples or across all samples. Importantly, it can accommodate the large number of missing phenotypic information within the dataset.

**Table 1:** Phenotype overview of four first-line drugs

	INH	RIF	EMB	PZA	4 1st-line
R	3361	2962	1558	1076	–
S	5393	5284	10515	3031	–
Tot.	8754	8246	12073	4107	3574

Note: R and S denote resistant and susceptible class, respectively. 4 1st-line means the four first-line drugs together i.e. INH, RIF, EMB and PZA.

## 2 Materials and data

### 2.1 Phenotyping

Our dataset includes 13 402 isolates that were tested to up to 11 drugs, including four first-line drugs: INH, RIF, ethambutol (EMB) and pyrazinamide (PZA). The drug-susceptibility testing (DST) was performed in either liquid culture or on solid medium, or both. All lineages were represented in [10, 11]. The phenotype overview of four first-line drugs is shown in Table 1.

### 2.2 DNA sequencing and pre-processing

The details of DNA sequencing are provided in [10]. Nucleotide bases were called using standard filters on sequencing and alignment quality, as well as the number of reads for each base. After filtering, the nucleotide bases at certain positions that could not be called with confidence were denoted as null calls and were not used in our analysis. We applied the same pre-processing method as described in [12]. Each sample has different number of genetic mutations.

## 3 Methodology

We propose a heterogeneous graph attention network to predict AMR (HGAT–AMR) to deal with the heterogeneous genetic data with variable-length. Graph neural networks operate on graph-structured data and can effectively capture the structural information encapsulated in graphs and generate more effective embedding. The graph attention network is one of a major branch of graph neural networks [13–16]. Attention in deep learning [17] can be thought of as a vector of importance weights, encoding which elements of a vector are learned as being important within the function of the particular network i.e. it encodes those elements of a vector the network is ‘paying attention to’ in undertaking its task (such as classification). This section starts with the introduction of the inputs, building blocks and architecture for HGAT–AMR, and then introduces ML comparators.

**Figure 1:**
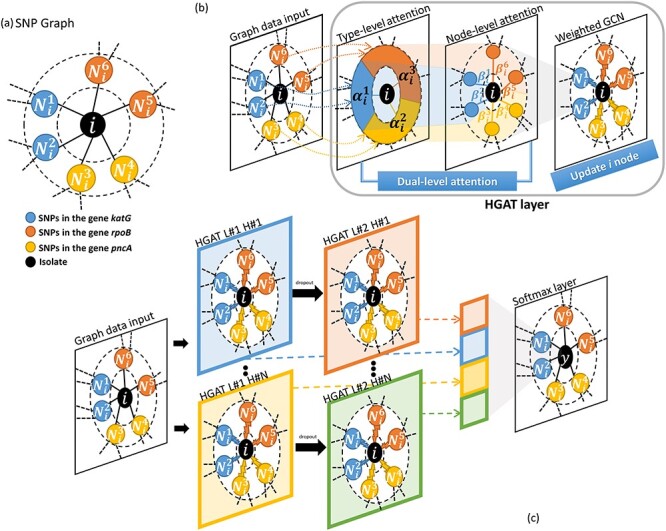
HGAT–AMR algorithm.

### 3.1 Construct input graph

The HGAT–AMR takes network-like data (i.e. a graph) as input, in which graph nodes correspond to isolates and to SNPs, and in which graph edges correspond to the relationship between the isolates and SNPs. Each node has different number of neighbouring nodes e.g. each isolate has different number of mutations, and each SNP occurs different number isolates. We term this undirected network-like input the ‘SNP graph’. In the MTB dataset, there are 24 types of node including: (i) isolates and (ii) SNPs of 23 types, where SNP nodes of the same type correspond to SNPs in the same gene. Figure 1A shows an example of SNP graph with an isolate node at the centre and its neighbours, which are SNPs found in three different genes, *katG*, *rpoB* and *pncA*. In this study, those 23 genes were investigated that constituted the assembled SNPs catalogue for the available tested drugs [11]. No edges were drawn between nodes of the same type as the intra-type relationship was unknown (e.g. the relationship between isolates, and between SNPs in the same gene). During training, the graph will be updated with edges between nodes in light of evidence within the training dataset, as described later.

### 3.2 Construct a HGAT module

For any given isolate, different mutations within different genes may carry information of varying importance when determining drug-resistance. To capture this hierarchical importance at both node—(SNP) and type—(gene) level, we applied a building block, the HGAT module [9], to be described below, which uses a ‘dual-attention’ mechanism within the graph (as shown in Figure 1B).

For an undirected and unweighted graph $G=(V,E)$, $v\in V$ and $e\in E$ represent nodes and edges, respectively. Let $X\in \mathbb{R}^{|V|\times q}$ be a matrix containing the features of nodes, and $x_v\in \mathbb{R}^q$ denotes features of the node $v$. If $G$ is homogeneous, the adjacency matrix is $A^{\prime}=A+I$ with added self-connections, the degree matrix is $M$, where $M_{i,i}=\sum _j A^{\prime}_{i,j}$, and the symmetric normalised adjacency matrix is $ \tilde{A}=M^{-1/2} A^{\prime} M^{-1/2}$. If $G$ is heterogeneous, where each node is associated with its types, $ v \in \tau _i$, so $V=\cup _{i=1}^m \tau _i $ with m types of nodes $ mathcal{T}=\{\tau _1,...,\tau _m \}$. Correspondingly, the type-wise adjacency matrix is $\tilde{A}_{\tau } \in \mathbb{R}^{|V| \times |V_{\tau } |}$ is a submatrix of $\tilde{A}$, whose rows represent all the nodes and columns represent their neighbouring nodes with the type $\tau $.

**Type-level attention** Given a specific node $v$, its hidden representation at the $l$th layer is $h_v^{(l)}$. Initially, $h_v^{(0)}=x_v$. The neighbouring node $v^{\prime}\in \mathcal{N}_v$ is with type $\tau $, whose hidden representation vector is $h_v^{\prime (l)}$. The hidden representation of the type $\tau $ is defined as $h_\tau ^{(l)}=\sum _{v^{\prime}} \tilde{A}_{vv^{\prime}} h_v^{\prime (l)}$, which is the sum of the neighbouring node of type $\tau $. Then, the type-level attention scores of the same layer are defined as,

(1) \begin{align*}& \alpha_\tau = \sigma(\mu_\tau^T \cdot [h_v^{(l)} || h_\tau^{(l)}]). \end{align*}

where $\mu _\tau $ is the attention vectors for the type $\tau $, $\parallel $ means concatenation and $\sigma (\cdot )$ denotes an activation function such as Leaky ReLU. The type-level attention weights are to normalise the attention scores across all the types with the softmax function,

(2) \begin{align*}& \alpha_\tau=\frac{\exp(a_\tau)}{\sum_{\tau\prime\in\mathcal{T}}{\exp(a_{\tau^\prime})}}. \end{align*}

**Node-level attention** Given a node $v$ with the type $\tau $, and its neighbouring node $v^{\prime}\in N_v$ with the type $\tau ^{\prime}$, the node-level attention scores of the $l$th layer are defined as,

(3) \begin{align*}& b_{vv\prime}=\sigma(\phi^T\cdot\alpha_{\tau\prime}[h_v^{(l)}||h_{v\prime}^{(l)}]). \end{align*}

where $\phi $ is the attention vector. The node-level attention scores are normalised with the softmax function,

(4) \begin{align*}& \beta_{v v\prime}=\frac{\exp(b_{v v\prime})}{\sum_{i \in \mathcal{N}_v}{\exp (b_{vi})}}. \end{align*}

As shown in Figure 1B, for the centre node $i$, the type-level attention is a vector of weights for different types of neighbours, e.g. $\alpha _i^1~\alpha _i^3$, where each type is a joint representation of all neighbours of the same type (such as the genes, or ‘colours’, within our graph). The node-level attention is a vector of weights for each neighbour e.g. $\beta _i^1~\beta _i^6$, after calibration using the type-level attention. Aggregately, the dual-level attention mechanism is incorporated into the graph convolution networks [18] module with the following layer-wise propagation rule:

(5) \begin{align*}& H^{(l+1)}=\sigma(\sum_{\tau\in\mathcal{T}}{\mathcal{B}_\tau\cdot H_\tau^{(l)}\cdot W_\tau^{(l)}}). \end{align*}

where $\mathcal{B}_\tau $ represents the attention matrix, whose element in the row $v$th and $v^{\prime}$th column are $\beta _{vv\prime }$, $W_\tau ^{(l)}\in \mathbb{R}^{q^{(l)}\times q}$ is type-wise transformation matrix and $H_\tau ^{(l)}$ is a hidden representation of neighbouring nodes of type $\tau $. Initially, $H_\tau ^{(0)}=X_\tau $.

### 3.3 Architecture

Based on the HGAT module, we constructed a deep graph learning model, HGAT–AMR (as shown in Figure 1C). In each layer, there are multiple different HGATs occurring in parallel, and, in keeping with the ML literature, each is named a head (analogous to the multiple ‘heads’ used in reading from/writing to tapes and disks in classical computer science). Each head of a layer was assumed to learn the different ‘aspects’ or ‘tracks’ of the input with different associated model values and attentions. This model structure was considered to account for the fact that there might be different mechanisms for single- and multi-drug-resistance within our AMR application. We used a two-layer model, as shown in Figure 1C, for the purpose of illustration. As shown, each head contains a two- connected HGAT, where the output of the first block undergoes ‘drop-out’ randomly (i.e. application of a random mask, as is common practice with deep learning) before being passed as input to the second one. Before the final layer, we concatenated the outputs of the second layer and the first layer, thus providing a ‘shortcut’ that connects the first layer and the final layer. (That is, the final layer sees both the outputs of the second layer and the corresponding outputs of the first layer, where the latter acts as a ‘shortcut’.) Formally,

(6) \begin{align*}& H^L=concat([H_{1}^{\left(i\right)}||\ldots||H_D^{\left(i\right)}]),i={1,...L-1}. \end{align*}

where $D$ denotes the number of the heads. These shortcut and multi-headed approaches are used in deep learning to avoid numerical stability problems during model training, such as ‘gradient explosion’ (in which some components of the network unduly dominate others) or ‘vanishing gradients’ (in which the models become so deep that the model cannot associate small changes in model parameter values with changes in performance), but come at the expense of increasing computational costs.

### 3.4 ML comparators

**Figure 2:**
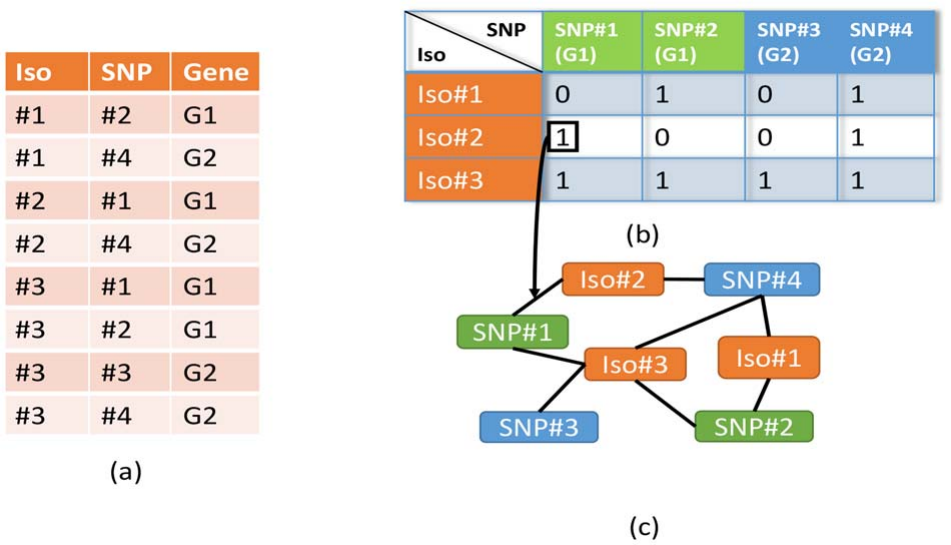
Illustration of input for discussed models.

This study considers the current clinical standard (direct association, DA) and several conventional ML methods. The details of the definition and implementation of the DA were the same as described in Yang et al. [12] and which form a useful ‘real-world’ comparator. The ML comparator models included, as noted above, support vector machines, logistic regression and multi-label random forests [19]. These methods were selected as being representative as the current state-of-the-art. Unlike HGAT–AMR, both the DA and conventional ML methods take as input a binary vector per sample, named grid data. Figure 2 illustrates how to convert tabular raw data into network-like input and grid input, respectively. The raw genetic data are illustrated in Figure 2A, which shows that three isolates (indexed by Iso1-3 as shown in the first column) have four SNPs (indexed by SNP#1-4 as shown in the second column), who belong to two genes (i.e. two types of SNPs denoted by G1 and G2 as shown in the third column). On the one hand, the grid input represents an isolate as a vector where each element corresponds to the presence (1) or absence (0) of a SNP (as shown in Figure 1B); the types of SNPs are not considered. On the other hand, the network-like input constructs a graph for the full tabular data, where the nodes’ colours denote their types. For example, orange corresponds to isolate, blue is SNPs of gene G2, and green is SNPs of gene G1. The SNP nodes’ types implicitly model the information of genes (as shown in Figure 1C).

## 4 Experiment settings

### 4.1 Initialisation

The initialisation of HGAT–AMR involves two components: (i) initialising SNPs’ embedding and (ii) initialising isolates’ embedding.

**SNPs’ embedding initialisation.** We compare genetic samples and mutation features to sentences and words in natural language, respectively. In the MTB dataset, we consider SNPs as equivalent to words and isolates (comprising SNPs) as equivalent to sentences (comprising words). Like word embedding in natural language processing, we first compute the SNP co-occurrence matrix based on point-wise mutual information (PMI). Then, we factorise the matrix using sparse singular value decomposition. The result of this procedure is an embedding vector of fixed size for each SNP, where every element is continuous (please see parameters of PMI-based embedding in Supplement B). We note that this embedding vector for a SNP comprises real (scalar) numbers, it being the result of factorising a matrix of PMI values (themselves real numbers).

**Isolates’ embedding initialisation.** The vector for each isolate is the aggregation of the embedding vectors for all of the SNPs present in the isolate. Naturally, the number of SNPs within an isolate will vary between isolates, and so the aggregator will accordingly take varying numbers of embedding vectors for different isolates. Common choices for the aggregator functions used to combine the multiple embedding vectors for the isolate’s SNPs into a single vector to represent that isolate include (i) the average, (ii) minimum, or (iii) maximum. In this study, we used an aggregator function of the maximum to initialise these representatives ‘feature vectors’ for each isolate node in the input graph. The evaluation of the other aggregator functions could be explored in future work, although our experiments are largely insensitive to such a choice.

### 4.2 Training models

**ML comparators.** For each model, the phenotypes to individual drugs for the isolates were used for classification in single-label learning, whereas those for multiple drugs were used for classification in multi-label learning. The conventional ML models were optimised via conventional cross-validation methods by choosing the values of the various hyperparameters using grid search. All comparator ML methods were trained in a conventional supervised manner (Please see Supplement C for the details, which represent standard practice for such classes of algorithms.)

**HGAT–AMR.** The output of Lth layer of HGAT–AMR, the embeddings of isolate nodes are then fed to a softmax layer for classification.

(7) \begin{align*}& Z=softmax(H_{isolate}^{(L)}). \end{align*}

During the training, the cross-entropy loss over training data with the L2-norm is applied.

(8) \begin{align*}& \mathcal{L}=\sum_{k=1}^{K}\sum_{i\in D_{train}}\sum_{j=1}^{C}{Y_{ij}^k\cdot l o g Z_{ij}}+\eta||\Theta||_2, \end{align*}

where $K$ is the number of multiple labels, $C$ is the number of classes, $D_train$ is the set of genetic samples for training, $Y$ is the corresponding label indicator matrix, $\Theta $ is the model parameters, and $\eta $ is regularisation factor. For model optimisation, we adopt the Adam optimiser. Two training modes were considered: (i) transductive and (ii) inductive. The former is standard supervised learning, whereas the latter is novel for our work and is the development of semi-supervised learning. In our MTB dataset, as noted earlier, isolates are frequently not tested for resistance to all relevant anti-TB drugs. The conventional way to cope with missing classes of the samples is to remove all such samples, or alternatively to split the unlabelled and labelled samples for conventional semi-supervised learning. The latter involves switching between ‘supervised’ and ‘unsupervised’ learning according to whether or not a sample (in our application, an isolate) has a complete set of class labels (in our application, DST results for all drugs). However, it typically produces substandard results in applications where there are few complete data (as in our MTB case), because unsupervised learning disregards any labels that might be available in the incomplete-data case. We improve upon this method for our HGAT–AMR model as described below in a manner that permits us to capitalise on all available labels, independent of their level of completeness (please see Supplement D).

## 5 Results

**Subsets for the supervised/transductive learning.** We first constructed four different subsets of our database for individual drugs (i.e. INH, RIF, EMB and PZA), which were used for single-label learning; the HGAT–AMR model trained on these datasets is termed as ‘HGAT–AMRs’ (noting the ‘s’ suffix). We then constructed a fifth subset, where all samples have complete phenotypes of all four drugs, for multi-label learning; the HGAT–AMR model trained on this dataset is named ‘HGAT–AMRm’.

**Subsets for the inductive learning.** We reconfigured the subset for the above supervised multi-label learning by (i) randomly removing the phenotypes of drugs of interest in the existing training set and (ii) augmenting subsets (otherwise comprising completely-labelled data) with any available incompletely-phenotyped samples. The HGAT–AMR model trained in the first experiment is named ‘HGAT–AMRi’ (for ‘inductive’), whereas the second is name ‘HGAT–AMRi-E’ (for ‘Enhanced’). In our later results, we indicate the ratio of the removed phenotypes by the percentage following the HGAT–AMRi e.g. ‘HGAT–AMRi-10%.’

**Data split and cross-validation.** To evaluate various models, we split each subset described above corresponding to examined drugs into training (40%), validation (40%) and testing sets (20%). For multi-label learning, we performed stratified cross-validation, which ensures that the proportion of the two classes (resistant versus susceptible) for every label was approximately the same in each fold. For the ML baseline models, as noted earlier, cross-validation was performed to identify the optimal values of the hyperparameters via standard grid-search that led to the best classification accuracy (Please see details of the hyperparameter set used for grid-search in Supplement B). The DA method did not require training, it being a clinical ‘look-up table’. An early-stopping criterion was applied while training the HGAT–AMR model, such that when the best validation accuracy of the model ceased increasing for at least ten training epochs, training was stopped to avoid over-fitting. For all methods, 20 iterations of the experiments (of 5-fold CV) were carried out, with the average performance of the resulting model being applied to the held-out testing dataset reported, as is conventional practice.

### 5.1 Prediction performance

Before going on to discuss the advantages offered by our proposed method, we first consider the classification task, in which each model aims to classify resistance/susceptibility for individual drugs. Table 2 shows the results of using eight models for predicting phenotypes of the four first-line drugs individually. The average (standard deviation) of sensitivity, specificity, F1-score and AUROC are reported on held-out data over the 20 iterations described above.

**Table 2:** Comparison of prediction for four first-line drugs

	INH				RIF			
Models	Sen	Spec	F1	AUROC	Sen	Spec	F1	AUROC
DA	94.05(0.78)	97.76(0.41)	96.11(0.40)	95.90(0.43)	96.29^*^(0.77)	97.66(0.41)	96.84(0.43)	96.97(0.45)
LR	93.85(0.71)	97.91(0.42)	96.12(0.40)	98.51(0.26)	94.92(0.91)	98.66(0.38)	97.07(0.43)	99.03(0.28)
SVM	93.95(0.75)	97.81(0.41)	96.10(0.39)	98.00(0.35)	94.79(1.06)	98.87^*^(0.33)	97.17^*^(0.45)	98.92(0.31)
MLRF	93.57(1.19)	97.83(0.87)	95.48(0.68)	98.18(0.44)	92.58(1.43)	97.19(0.72)	94.17(0.92)	98.33(0.45)
HGAT–AMRs	94.46(0.95)	97.76(0.47)	96.28(0.42)	98.33(0.33)	95.17(1.11)	98.50(0.44)	97.05(0.45)	99.10^*^(0.21)
HGAT–AMRm	94.74(1.16)	98.32^*^(0.64)	96.60(0.63)	98.43(0.45)	95.21(1.78)	97.29(1.24)	96.30(1.19)	98.99(0.39)
HGAT–AMRi-10%	94.78(1.12)	98.13(0.61)	96.52(0.59)	98.43(0.42)	94.92(1.44)	95.86(1.89)	95.33(1.41)	98.76(0.44)
HGAT–AMRi-E	94.91^*^(1.06)	98.20(0.60)	96.62^*^(0.57)	98.53^*^(0.35)	93.39(1.37)	94.39(1.93)	94.02(1.44)	98.32(0.57)
	EMB				PZA			
Models	Sen	Spec	F1	AUROC	Sen	Spec	F1	AUROC
DA	84.90(1.85)	94.91(0.47)	86.86(1.03)	89.91(0.99)	50.65(3.65)	95.02^*^(0.84)	75.43(1.90)	72.83(1.86)
LR	76.38(2.37)	97.90^*^(0.36)	87.37^*^(0.92)	96.68^*^(0.56)	74.15(2.78)	92.67(1.04)	83.95^*^(1.16)	93.70^*^(0.82)
SVM	75.30(3.00)	97.08(0.49)	86.96(0.99)	95.83(0.67)	72.68(3.10)	92.89(1.64)	83.55(1.45)	92.91(1.01)
MLRF	74.71(3.66)	90.79(1.35)	74.96(2.39)	93.17(0.87)	69.97(3.66)	91.93(1.26)	72.41(2.43)	92.61(0.87)
HGAT–AMRs	79.45(3.90)	96.31(0.64)	87.16(1.04)	96.50(0.46)	73.59(4.52)	91.10(1.66)	82.43(1.57)	92.21(0.94)
HGAT–AMRm	82.13(5.50)	88.97(2.34)	84.25(1.42)	93.88(0.80)	76.56(5.08)	89.53(1.93)	82.30(1.59)	92.80(0.89)
HGAT–AMRi-10%	92.76(2.62)	84.33(1.71)	84.64(1.30)	93.99(0.83)	89.79(3.89)	84.26(2.31)	83.08(1.64)	92.90(1.01)
HGAT–AMRi-E	96.60^*^(1.47)	79.50(2.04)	82.45(1.55)	93.62(0.90)	90.63^*^(2.75)	83.20(2.68)	82.58(1.77)	92.52(0.92)

Note: The P-value of performance measurement in bold compared to DA was obtained by Wilcoxon signed-rank test. ^*^ represents a P-value<0.05.

Considering general classification performance, the ML methods outperformed the DA approach for all four drugs except for sensitivity in RIF and specificity in PZA. In the cases of INH, EMB and PZA, the best sensitivities were obtained by the HGAT–AMRi-E (i.e. 94.91, 96.60 and 90.63%, respectively). For INH and RIF, the best AUROCs were obtained by two of the HGAT–AMR models, 98.53% by HGAT–AMRi-E and 99.10% by HGAT–AMRs. For the EMB and PZA, the LR generated the best F1-score of 87.37 and 83.95% and the best AUROC of 96.68 and 93.70%. In terms of specificity, the best values were 98.32% of HGAT–AMRm for INH, 98.87% of SVM for RIF, 97.90% of LR for EMB and 95.02% of DA for PZA,. The HGAT–AMRs showed similar results as the LR, especially in the cases of EMB and PZA. This is because they were trained in the same mode i.e. supervised single-label learning. The multi-label learning approach enabled the HGAT–AMR model to increase sensitivity by 0.2% for INH and RIF, and by 3% for EMB and PZA. This could be explained by the genetic patterns corresponding to the resistance co-occurrence that are predictive for these cases. Furthermore, inductive learning allowed the HGAT–AMR models to continue the increment in sensitivity for INH, EMB and PZA, but to decrease for RIF. The inductive learning essentially loosened the dependence of resistance co-occurrence and resistance prediction by introducing incomplete phenotypes for all examined drugs. It implies that the resistance to all four first-line drugs except for RIF could be predicted better if the evidence of resistance co-occurrence is relaxed. For INH and RIF, the differences in all performance measurements across different methods were less than 4%. For EMB and PZA, the significant improvement in sensitivity compared to the DA was obtained by HGAT–AMRi-E, which were 12 and 41%, respectively. This can be explained by (i) associations between SNPs and the resistance to INH or RIF being nearly linear, and this is consistent with the results obtained by the DA method, (ii) associations between SNPs and the resistance to EMB or PZA are more complex and (iii) patterns of resistance co-occurrence mask those SNPs associated with EMB or PZA-resistance.

### 5.2 Attention interpretation

A key advantage of the proposed method, beyond any (modest or otherwise) improvements in classification performance is the interpretable nature of the resulting graph constructed during the training of the HGAT. For the purposes of demonstration and computational efficiency, we applied two-head attention in the cases of INH, RIF and EMB, and four-head attention for PZA. The reason for using four heads with PZA is that the use of two heads was experimentally (not shown here for brevity) found not to capture the resistance patterns associated with PZA, which was assumedly masked by patterns of resistance co-occurrence. Therefore, there are four sets of type-level and node-level attention scores for each node for the two-headed models (INH, RIF, EMB) and eight sets of attention scores for the four-headed model (PZA). We index attention scores by head and layer: for example, ‘1L1H’ refers to the 1st head of the 1st layer.

**Figure 3:**
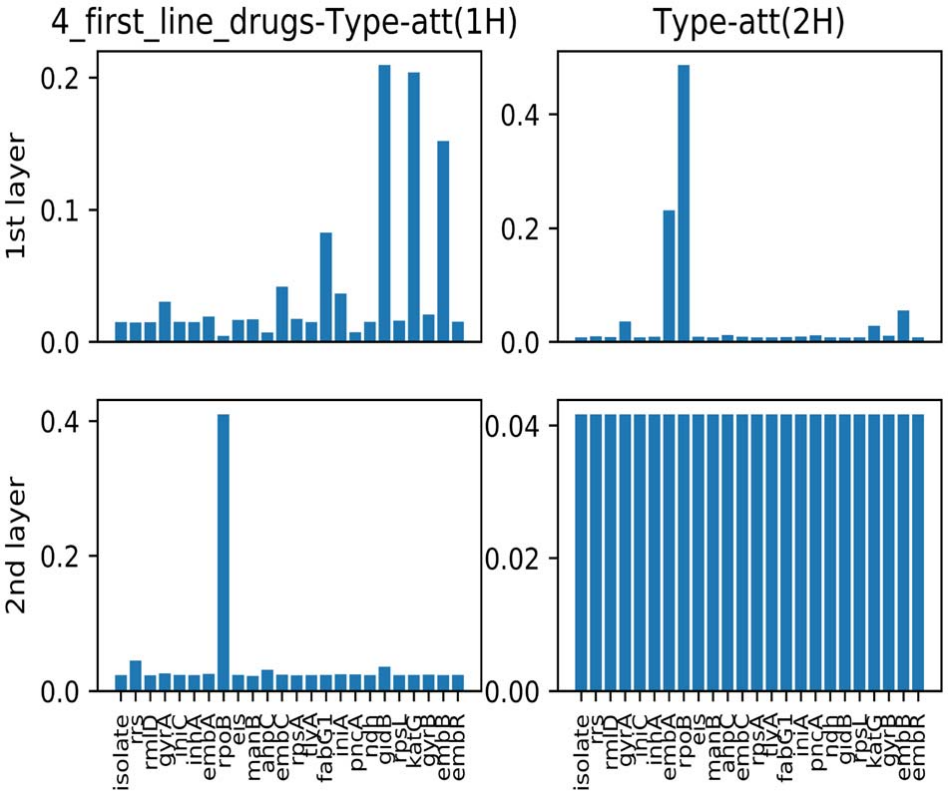
A demonstration of averaged type-level attention scores for all four first-line drugs.

**Averaged type-level attention scores.** Figure 3 demonstrates the averaged type-level attention scores obtained via multi-label learning across all training isolates. Considering all four first-line drugs jointly, the first head of the first layer recognised gidB, katG and embB as the most interpretable genes, and that of the second layer further identified rpoB to be the only dominant gene. In contrast, the top two genes ranked by the second head of the first layer were rpoB and embA. It is notable that the PZA-associated gene, pncA, is not shown in Figure 2. This can be explained by (i) the genes being associated with the other three drugs strongly dominant for the joint drug-resistance prediction, and contributing to PZA-resistance prediction due to drug-resistance co-occurrence; thus, the contribution of pncA was masked and (ii) the samples resistant to PZA being relatively fewer than others.

The plots of averaged type-level attention scores for each drug via single-label learning are referred in Figure S3 of Supplement E. For INH (as shown in Supplement E Figure S3A), the top two genes ranked by both the two heads of the first layer were the two main INH-associated genes, katG and fabG1, whereas both the two heads of the second layer considered all genes to be equivalent. It is also observed that all genes were scored the same by the second layers in the case of EMB and PZA, and the second head of the second layer when considering the four drugs jointly. For RIF (as shown in Figure S3B), all heads and layers identified the main RIF-associated gene, ropB. This can be explained by the gene of rpoB being sufficiently obvious to be identified by all layers, and being sufficient for predicting RIF-resistance. In Figure S3C, the top three genes ranked by the first layer were embB, embA and katG. The first two genes were EMB-associated genes but scored differently by two heads. The gene of katG was also recognised due to resistance co-occurrence between INH and EMB. In the case of PZA (Figure S3D), the first layer barely recognised any particular genes. On the contrary, the second head of the second layer identified katG and rpoB, which were associated with INH and RIF resistance, respectively. This could be because the drug-resistance co-occurrence patterns for INH and RIF are also predictive for PZA resistance. Unlike the second head, the third head identified pncA only, which is the key PZA-resistance-related gene. It can be explained that different heads discovered different ‘aspects’ or ‘tracks’ of genetic mutations to predict PZA-resistance. Moreover, the different patterns of attention scores across layers for different drugs also imply that genetic variables contribute to drug-resistance prediction with different ‘depth’.

**Figure 4:**
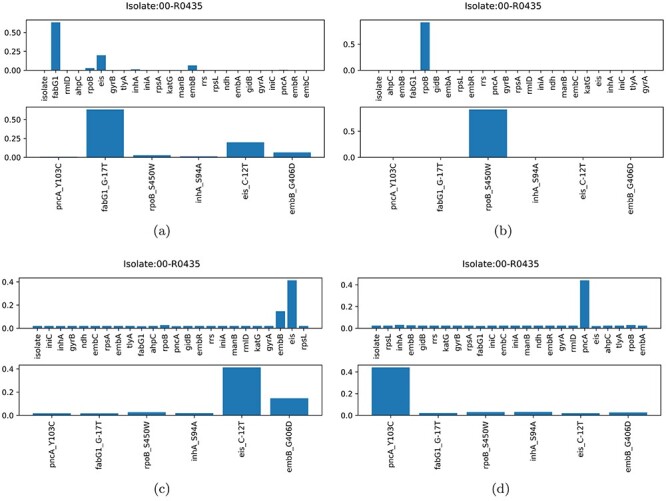
The isolate ‘00-R0435’: averaged isolate-level attention scores for (**A**) INH, (**B**) RIF, (**C**) EMB and (**D**) PZA.

**Node-level attention scores.** Figure 4 demonstrates the node-level attentions for an exemplar isolates whose ids is 00-R0435, who were resistant to all four first-line drugs according to the DST. In the case of INH, the model ranked *fabG1_G-17T* as the top-1 SNP, which was previously identified as being associated with INH resistance (as shown in Figure 4A). Similarly, only *rpoB_S450W* was identified in the case of RIF, which was previously identified as being RIF-resistance-associated SNPs (as shown in Figure 4B). In Figure 4C, the top-two SNPs are *eis_C-12T* and *embB_G406D* for predicting resistance to EMB, where the latter was previously identified to be related to EMB-resistance. In the case of PZA, 2L3H identified a SNP in the gene of pncA, *pncA_Y103C*. It is worth noting that each head would identify different SNPs given the type-level attention scores for an individual isolate. Supplement E, Figure S4 provides another example for demonstrating SNP-level attention.

### 5.3 SNPs ranking

Another key advantage of the proposed method is in identifying structural associations between nodes in the graph and the corresponding phenotypic labels. Table 3 lists the top-10 SNPs ranked by averaged node-level attention scores with respect to each drug, and to all first-line drugs jointly. Those previously-reported SNPs for specific drug-resistance are represented in bold. The presence of these highlighted items is encouraging, as it represents the graph structure capturing those associations that are known in the literature. For individual drugs, the different heads of the same layer often generate a different list of interpretable SNPs, which again validated that the model ‘sees’ different aspects of the pattern for the prediction task. It is notable that the number of heads of attention can be increased (as described above, with our four-headed model for PZA) to investigate different aspects of the input. Specifically, the seven of the top-ten SNPs of pncA were previously recognised as associated with PZA-resistance in the existing clinical (DA) catalogue. For the four first-line drugs as a whole, the top-ten SNPs ranked by the first layer include those relating to resistance of INH and EMB, whereas the second layer paid more attention to those related to RIF-resistance. This is also consistent with averaged type-level attention in Figure 3B, where the gene of rpoB was sufficiently robust to be identified by both of the two layers.

**Table 3:** List of the top-ranked SNPs for individual drug and all four first-line drugs jointly

INH		EMB		
1st-L 1st-H	1st-L 2nd-H	1st-L 1st-H	1st-L 2nd-H	2nd-L 3rd-H
**’katG_S315T’**,	**’katG_S315T’**,	**’embB_M306V’**,	**’embB_M306I’**,	’pncA_A102P’,
**’katG_S315N’**,	**’katG_S315N’**,	**’embA_C-12T’**,	’katG_I248T’,	’pncA_L4W’,
**’fabG1_C-15T’**,	**’fabG1_C-15T’**,	**’embA_C-16T’**,	’embA_G-76C’,	**’pncA_V139L’**,
’ahpC_C-54T’,	**’fabG1_G-17T’**,	**’embB_Q497R’**,	’embA_Q1004P’,	**’pncA_C14R’**,
**’fabG1_G-17T’**,	’gyrA_G249G’,	**’embB_M306I’**,	**’embB_Q497R’**,	**’pncA_M175V’**,
**’fabG1_L203L’**,	’ahpC_C-54T’,	’embA_G-76C’,	’katG_D448A’,	’pncA_V130G’,
’gyrA_G249G’,	**’fabG1_L203L’**,	’embA_Q1004P’,	**’embA_C-16T’**,	**’pncA_V139G’**,
’ahpC_G-48A’,	’rpoB_Q432K’,	’embA_C-8A’,	’embA_C-12T’,	**’pncA_V155G’**,
’gyrA_D639A’,	’ahpC_G-48A’,	**’embB_G406S’**,	’embA_E951D’,	**’pncA_489_delC’**,
**’fabG1_T-8C’**	’gyrA_V374V’	’embA_E951D’	’embA_C-8A’	**’pncA_V21G’**
RIF			Four first-line drugs	
1st-L 2nd-H	1st-L 2nd-H	2nd-L 1st-H	1st-L 1st-H	2nd-L 1st-H
rpoB_V553V’,	**’rpoB_S450L’**,	’rpoB_L731P’,	**’fabG1_C-15T’**,	’rpoB_A286V’,
’rpoB_S4S’,	**’rpoB_D435V’**,	’rpoB_A692T’,	’iniA_D549E’,	’rpoB_R827C’,
’rpoB_S1133S’,	’rpoB_P471P’,	’rpoB_I1106T’,	’gyrA_D641E’,	’rrs_C1474T’,
**’rpoB_V359A’**,	**’rpoB_H445Y’**,	’rpoB_Q409R’,	**’embB_M306V’**,	’rpoB_F503S’,
’rpoB_S431G’,	’rpoB_S1133S’,	’rpoB_R827C’,	**’fabG1_G-17T’**,	’rrs_G368C’,
’rpoB_G-21C’,	’rpoB_V168A’,	’rpoB_S428R’,	**’katG_S315T’**,	**’rpoB_I491F’**,
’rpoB_H749Y’,	’rpoB_S4S’,	**’rpoB_V170F’**,	**’embB_M306I’**,	’rpoB_L430R’,
’rpoB_G876G’,	**’rpoB_S450W’**,	**’rpoB_H445D’**,	’gyrA_D94G’,	’rpoB_E481A’,
**’rpoB_M434I’**,	’rpoB_L430R’,	**’rpoB_H445C’**,	’iniA_N88S’,	**’rpoB_H445L’**,
’rpoB_L430R’	’rpoB_V553V’	’rpoB_S431G’	’gyrB_V210V’	**’rpoB_H445C’**

Note: ‘L’ and ‘H’ denote layer and head, respectively.

### 5.4 Inductive learning evaluation

**Figure 5:**
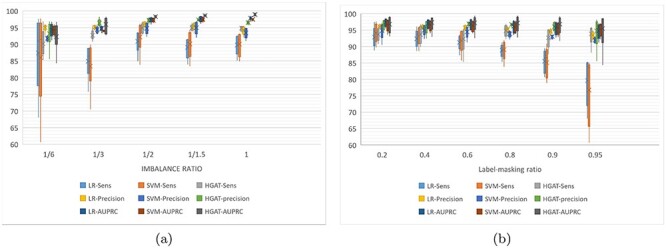
Inductive training performance of HGAT of for INH.

**Figure 6:**
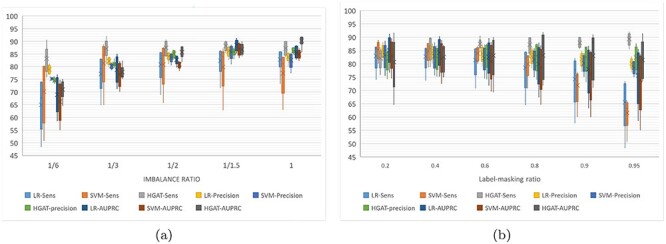
Inductive training performance of HGAT of for PZA.

**Figure 7:**
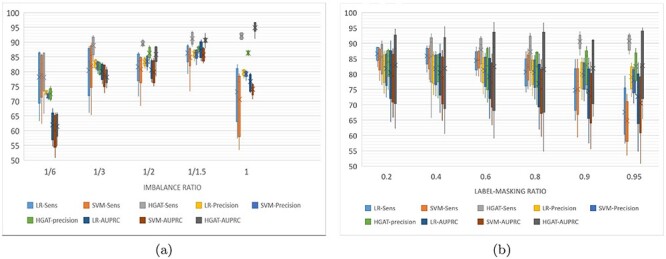
Inductive training performance of HGAT of for EMB.

This section evaluates the ability of HGAT–AMR to deal with incomplete labels. We reconfigured the training set for transductive learning (trans-training set) by removing the phenotypes gradually, as outlined earlier. During the training, the phenotype of each of the four drugs could now be absent. For example, an isolate could have been tested by INH and EMB, but not by RIF and PZA. According to an imbalance ratio for a specific drug, the trans-training set is resampled to construct a new training set. The label-masking ratio gradually increased on each resampled training set, and the model is trained for 100 times at each label-masking ratio. The experiment compared baseline models (LR, SVM) and HGAT. The examined models are verified on the same test dataset with a class-imbalance ratio and label-masking ratio. Performance evaluation measurements include sensitivity, precision and AUPCR. It is worth noting that the baseline model is trained with a single-label learning framework, whereas HGAT is trained with multi-labels learning framework. Besides, the optimal threshold for calculating sensitivity and precision is determined by AUROC. Figures 5–7 are box-and-whisker plots of the prediction performance for INH, PZA and EMB, respectively. The performance of the RIF and INH models is similar. Please see Tables (S4–S7) for detailed results. Figure 5A shows that HGAT outperforms LR and SVM, except when the data are highly imbalanced (class-imbalance ratio (R:S) is 1:6). When the R:S ratio is 1:3, HGAT’s area under the receiver operating characteristic (AUPRC) has large interquartile range (IQR) (Q3-Q1); the AUPRC of HGAT is as low as 84.4% at the high label-masking ratio (0.95). This may be explained by the fact that the co-resistance related pattern inferred by HGAT introduces errors in predicting INH-resistance at high label-masking ratio. In Figure 5B, LR and SVM’s sensitivity decreases as the training set shrinks to 80% or less, whereas HGAT remains at about 95%. This shows that when predicting INH resistance, HGAT performs better when the label-masking ratio is high. In Figure 6, the sensitivity of HGAT is always higher than that of LR and SVM. At the class-imbalance ratio of 1:6, 1:2 and 1:1, HGAT’s AUPRC is better than LR and SVM. At the ratio of 1:6 and 1:3, the precision of LR is higher. This means that HGAT classifies less PZA-resistant samples to be susceptible, whereas LR did better the other way around; the former has a more significant error clinically. In Figure 6B, LR and SVM’s performance decreases with the increase of the label-masking ratio, and the sensitivity is as low as 60%. The sensitivity of HGAT is always above 85%, and the precision and AUPRC remain unchanged. This shows that HGAT is robust when training with incomplete labels. In Figure 7A, HGAT’s AUPRC is better than the other two methods except when the data are relatively unbalanced (1:6 and 1:3). At the unbalanced ratio of 1:3, the sensitivity of HGAT is higher than that of LR and SVM. When the data are balanced, the average values of HGAT’s sensitivity, precision and AUPRC are 93, 86 and 95%, respectively (nearly 15, 5 and 15% higher than the other two methods, respectively). Figure 7B shows that the sensitivity of HGAT slightly increases, but AUPRC remains unchanged whereas the label-masking ratio reduces. In comparison, LR and SVM’s sensitivity decreases from 86 to 66%, and the AUPRC decreases from 80 to 73%. The IQR of AUPRC obtained by HGAT is relatively large, meaning that AUPRC varies considerably over the different imbalance ratio. This can be explained by the fact that the co-resistance feature is not advantageous for HGAT when the data are strongly imbalanced.

## 6 Discussion

### 6.1 Performance explanation

In the evaluation of prediction performance, HGAT–AMRi-E outperforms the others in terms of sensitivity for all four drugs except RIF. This could be because the extra subset enables the model to learn more patterns from data with incomplete phenotypes. For RIF, the DA and conventional ML models outperform the proposed models in sensitivity, specificity, and F1-score. This can be explained by (i) ropB being the only gene related to RIF-resistance among the considered genes and (ii) that most of the RIF-resistance-associated SNPs in rpoB have been recognised. In contrast, the proposed model considers all genes and the performance was only lower than the best sensitivity, specificity, and F1-score by up to 1% for RIF. This also testifies to the fact that the attention mechanism of the model is able to capture the RIF-associated genes (equivalent to the type as described in the model) and SNPs (equivalent to the node as described in the model). Overall, the sensitivity was increased at the expense of reducing specificity. F1-score is a harmonic mean of precision and recall. In the cases of EMB and PZA, the F1-score is lower because the sensitivities were lower and the trade-off between sensitivity and specificity was larger than the cases of INH and RIF. This can be explained by (i) the genetic pattern of drug-resistance co-occurrence dominating the prediction of EMB and PZA, (ii) the possible underestimation of the effect of predictive SNPs associated with EMB and PZA or (iii) a greater preponderance of phenotypic errors for EMB and PZA.

### 6.2 Explanation of dual attentions

The layer-wise type-level averaged attention scores are able to rank the interpretable genes for different drug-resistance predictions. The different attention heads capture different aspects of the predictive genetic patterns. These scores would enable researchers to (i) shortlist effectively the candidates of genetic variables associated with specific drug-resistance by looking into those highly ranked genes and (ii) efficiently identify the candidates of contributed genes given all mutations, especially when predicting drug-resistance without knowing the genes associated with less-studied drugs. Furthermore, the pattern in the layer-wise type-level attentions can be helpful in interpretating genes when the isolates exhibit resistance co-occurrence. For example, all layers and all heads that captured the gene rpoB might explain the high probability that this gene is the only RIF-resistance-associated gene, and the pattern of resistance co-occurrence could minimally influence the RIF-resistance prediction. Conversely, the EMB and PZA-resistance prediction could be highly affected by considering whether the genetic pattern corresponds to resistance co-occurrence.

### 6.3 Ranked SNPs

As with conventional feature selection in LR and RF methods, we equivalently generated SNP ranking lists by averaging node-level attention scores. Since the PMI initialisation has removed the most frequent and least frequent words in the corpus (SNPs in all samples), the proposed model will not consider those most and least common SNPs, which aids in the discovery of informative SNPs beyond the most dominant. With our two-level attention structures, the proposed model was inclined to identify the SNPs from the most dominant genes identified by the type-level attention. In the case of INH and EMB, the SNPs associated with resistance were ranked higher by the first layer. For RIF, both the two layers captured several subgroups of RIF-resistance-associated SNPs. For PZA, only 2L3H identified PZA-resistance-associated SNPs, whereas the other attentions captured the SNPs associated with other drugs due to drug-resistance co-occurrence. This list is consistent with the type-level attention scores in Figure 2. It is worth noting that increasing the number of heads would allow discovery of more hidden patterns. This is because the two-headed model, used for INH, RIF and EMB, cannot identify the gene pncA, whereas the model was able to capture this gene when its heads increased to four. We acknowledge that conventional ML models along with the subset of selected SNPs could improve the performance of prediction if that is the primary factor of interest. Such subsets could include the most predictive SNPs, although they may belong to genes that are unlikely to contribute to resistance acquisition for the considered drug. Here, we provide a way to rank the predictive SNPs from different heads, which improves the model’s interpretability by ”viewing” the drug-resistance co-occurrence through different viewpoints learned by the various heads.

### 6.4 Explanation of ‘transductive’ and ‘inductive’

Transductive learning is equivalent to standard supervised learning, where each sample (i.e. the nodes in graph model) in the training set has complete annotations. The inductive learning is semi-supervised learning, where not all samples of the training set have annotations and the unlabelled samples are considered to be ‘silent’ for classification. In the framework of multiple label learning, an advantage of the inductive learning would be that each sample in the training set is allowed to have incomplete annotations for all labels. In the context of multiple drug-resistance predictions, the ‘inductive’ training allows the isolates to have phenotypes missed randomly for drugs. As we have discussed, it is common to have incomplete phenotype annotations with respect to the full spectrum of drugs. The inductive learning method allows those samples with incomplete phenotypes to be used as much as possible and reduce information loss. It can be generalised for less tested drugs. For certain drugs, the predictive influence of resistance co-occurrence can be mitigated by randomly removing the phenotype of different drugs. In the case of INH, when the data are highly imbalanced (1:6), the three models’ precision and AUPRC decrease, but the mean and median remain higher than 90% (as shown in Figure 4A). Meanwhile, the three models’ precision and AUPRC maintains at about 95% as the label-masking ratio increases (as shown in Figure 4B). This may be because there are very few genetic factors that distinguish INH-resistant samples from non-resistant samples. For example, if we do not consider the known INH-resistance-associated genes, the sensitivity drops to 50%, and the precision is still higher than 90%. In the case of PZA, it is worth noting that the original data’s class ratio for PZA was around 1:3. In the experiment, we down-sampled susceptible samples to make the data more balanced and down-sampled resistant samples to have more imbalanced data. It implies that (i) adding PZA-susceptible samples helps improve the performance of HGAT, but not for LR and SVM and (ii) reducing PZA-resistant samples hurts all three models. In the case of EMB, all three methods’ performance is low when the imbalance ratio is 1:6 (as shown in Figure 6A). It suggests that severe imbalance of data harms both single-label and multi-label learning models. The HGAT outperforms the other two models when the data are balanced. This may be because HGAT is enhanced by multi-label learning, and the inferred co-resistance pattern helps the prediction of EMB-resistance.

### 6.5 Limitations

The proposed model uses full gradients of all nodes and the entire adjacency matrix within the graph, and so one of its limitations is high computational cost. It requires more efficient methods than those used here to deal with whole-genome data and much larger datasets. In this study, we only applied up to three head attentions in each layer due to the computational cost. We demonstrated these scores on a new training set by combining the original training and testing datasets, which could lose the information in the validation dataset. Meanwhile, because the initialisation was carried on the entire dataset in an unsupervised manner, this might be problematic if the nodes were unseen in the training set. This could be resolved by extending the model to the generative model.

## 7 Conclusion

In this work, we proposed HGAT for predicting MTB drug resistance. By considering a different number of genetic variables per genetic sample and the relationship between mutations and corresponding genes, the HGAT can model genetic samples with more interpretability. The dual-level attention scores offer a better insight for understanding the resistance mechanism from the levels of mutations and genes for different anti-TB drugs. The identified drug-resistance-related genes are correctly identified for the examined anti-TB drugs. The most top-ranked mutations are consistent with domain knowledge. The multi-label optimisation scheme coupled with graph learning enables the model to learn from the data with incomplete drug-resistance phenotypes. The experiment results show that the proposed model is more robust when the training data are highly imbalanced and when more phenotypes are missing.

## Data availability statement

Data are all already available in EBI/NCBI under the accession number(s) provided in [11].

## 8 Author contributions statement

Y.Y. and D.C. conceived the experiments, Y.Y. conducted the experiments, Y.Y., S.K, C.W, T.M.W, T.E.A.P and D.W.C. analysed the results. Y.Y. wrote the manuscript. Y.Y., S.K, T.M.W, T.E.A.P, D.W.C. and D.C, CRYPTIC Consortium, and other contributors reviewed the manuscript.

**CRYPTIC Consortium** Derrick W Crook, Timothy EA Peto, A Sarah Walker, Sarah J Hoosdally, Ana L Gibertoni Cruz, Joshua Carter, Sarah Earle, Samaneh Kouchaki, Yang Yang, Timothy M Walker, Philip W Fowler, Daniel Wilson and David A Clifton, University of Oxford; Zamin Iqbal, Martin Hunt and Jeff Knaggs, European Bioinformatics Institute; Daniela M Cirillo, Emanuele Borroni, Simone Battaglia, Arash Ghodousi, Andrea Spitaleri and Andrea Cabibbe, Emerging Bacterial Pathogens Unit, IRCCS San Raffaele Scientific Institute, Milan; Sabira Tahseen, National Tuberculosis Control Program Pakistan, Islamabad; Kayzad Nilgiriwala, Sanchi Shah, Ayan Mandal and Nerges Mistry, The Foundation for Medical Research, Mumbai; Camilla Rodrigues, Priti Kambli, Utkarsha Surve and Rukhsar Khot, P.D. Hinduja National Hospital and Medical Research Centre, Mumbai; Stefan Niemann, Thomas Kohl and Matthias Merker, Research Center Borstel; Harald Hoffmann, Katharina Todt and Sara Plesnik, Institute of Microbiology & Laboratory Medicine, IML red, Gauting; Nazir Ismail, Shaheed Vally Omar, Lavania Joseph Dumisani Ngcamu, Nana Okozi and Shen Yuan Yao, National Institute for Communicable Diseases, Johannesburg; Guy Thwaites, Thuong Nguyen Thuy Thuong, Nhung Hoang Ngoc and Vijay Srinivasan, Oxford University Clinical Research Unit, Ho Chi Minh City; David Moore, Jorge Coronel and Walter Solano, London School of Hygiene and Tropical Medicine and Universidad Peruana Cayetano Heredia, Lima; George F Gao, Guangxue He, Yanlin Zhao, Aijing Ma and Chunfa Liu, China CDC, Beijing; Baoli Zhu, Institute of Microbiology, CAS, Beijing; Ian Laurenson and Pauline Claxton, Scottish Mycobacteria Reference Laboratory, Edinburgh; Robert J Wilkinson, University of Cape Town, Imperial College London and Francis Crick Institute; Anastasia Koch, University of Cape Town; Ajit Lalvani, Imperial College London; James Posey, CDC Atlanta; Jennifer Gardy, University of British Columbia; Jim Werngren, Public Health Agency of Sweden; Nicholas Paton, National University of Singapore; Ruwen Jou, Yu-Xin Xiao, Wan-Hsuan Lin, CDC Taiwan; Lucilaine Ferrazoli, Rosangela Siqueira de Oliveira, Juliana Maira Watanabe Pinhata, Institute Adolfo Lutz, São Paulo. James Millard, Africa Health Research Institute, Durban Rob Warren - University of Stellenbosch, Cape town Annelies Van Rie, University of Antwerp Simon Grandjean Lapierre, Marie-Sylvianne Rabodoarivelo and Niaina Rakotosamimanana, Institut Pasteur de Madagascar Camus Nimmo, University College London Kimberlee Musser and Vincent Escuyer, Wadsworth Center, New York Ted Cohen, Yale University

**Those contributing to the CRyPTIC consortium:** Inaki Comas, Biomedicine Institute of Valencia Maha Farhat, Harvard University Jennifer Gardy, formerly BCCDC and now BMGF Vitali Sintchenko, University of Sydney Vanessa Mathys, Sciensano, Belgian reference laboratory for M. tuberculosis Dick van Soolingen, RIVM, Netherlands Philip Supply, National Center for Scientific Research, France Mark Wilcox, Leeds teaching Hospital NHS Trust Angkana Chaiprasert, Mahidol University, Bangkok, Thailand Irena Arandjelovic, University of Belgrade, Serbia

## Supplementary Material

Supplements-2021-4-8_bbab299

## Notes

**Yang Yang** is a Senior Research Associate in the Department of Engineering Science, University of Oxford, UK. Her research interests including digital health, health informatics, machine learning, computational biology and signal processing.

**Timothy M. Walker** is a Wellcome Trust Clinical Career Development Fellow based at Oxford University Clinical Research Unit, Ho Chi Minh City, Vietnam.

**Samaneh Kouchaki** is Lecturer in Machine learning for Healthcare at the Department of Electronic and Electrical Engineering and a member of the Centre for Vision, Speech and Signal Processing (CVSSP) at the University of Surrey. Her research interests include machine learning, health informatics, biomedical signal processing and computational biology.

**Chenyang Wang** received the BEng degree in Computer Science and Technology from Shanghai Jiao Tong University (SJTU), China, in 2019. He is currently a DPhil student in the Department of Engineering Science, University of Oxford, UK. His current research interest is machine learning in healthcare.

**Tim E.A. Peto** is a professor of medicine at the University of Oxford. He is the co-leader for the Infection Theme of the Oxford Biomedical Research Centre, and a National Institute for Health Research Senior Investigator.

**Professor Derrick Crook** is a Professor of Microbiology, Nuffield Department of Medicine, University of Oxford and Infectious Diseases Physician, Oxford University Hospitals NHS Trust.

**David A. Clifton** is a Professor of Clinical Machine Learning in the Department of Engineering Science, University of Oxford, UK and leads the Computational Health Informatics (CHI) Lab.
