# Supplementary material for: An end-to-end heterogeneous graph attention network for Mycobacterium tuberculosis drug-resistance prediction
Source: Brief Bioinform. 2021 Aug 20;22(6):bbab299. doi: 10.1093/bib/bbab299 (PMC8575050; doi:10.1093/bib/bbab299)
Supplement: Supplements-2021-4-8_bbab299 [file supplements-2021-4-8_bbab299.pdf]

Supplement A

In this supplement, we provide the details of graph for five MTB subsets in Figure S.1.

Figure S.1 Details of MTB graphs

**Parameter name**	**Value**
# types of nodes	24
Types of nodes	Isolate, embR, gyrB, inhA, ndh, rpoB, fabG1, rpsL, gyrA, embC, iniC, gidB, rpsA, tlyA, rmlD, rrs, manB, embB, embA, katG, iniA, ahpC, pncA, eis
**INH subset**	
# nodes of each type	[8754, 26, 62, 17, 25, 111, 18, 10, 101, 53, 21, 132, 21, 12, 17, 203, 13, 98, 83, 70, 37, 31, 130, 29]
# edges [isolates v.s. SNPs]	[0, 1352, 2446, 599, 791, 12354, 1239, 8152, 4662,13071, 409, 10209, 2829, 6703, 2398, 3830, 2549, 5018, 5885, 9859, 2412, 2706, 2586, 889]
**EMB subset**	
# nodes of each type	[12073, 36, 93, 16, 22, 113, 17, 13, 132, 82, 38, 137, 27, 14, 22, 237, 23, 121, 101, 66, 50, 31, 128, 33]
# edges [isolates v.s. SNPs]	[0, 1425, 3149, 589, 756, 13155, 1243, 11219, 5472, 18101, 497, 10816, 2878, 9873, 2652, 5947, 2596, 5741, 6104, 9359, 2594, 2662, 2549, 886]
**RIF subset**	
# nodes of each type	[8246, 24, 65, ,12, 21, 126, 14, 12, ,100, 48, 21, 119, 22, 13, 15, 185, 13, 89, 74, 49, 35, 27, 131, 28]
# edges [isolates v.s. SNPs]	[0, 1067, 1996, 525, 389, 13662, 1129, 8273, 4174, 13167, 344, 9828, 2702, 6847, 2035, 3302, 2518, 4551, 5415, 8922, 1567, 2575, 2591, 836]
**PZA subset**	
# nodes of each type	[4107, 12, 31, 9, 11, 71, 14, 10, 62, 26, 14, 58, 27, 6,12, 164, 7, 65, 48, 28, 16, 15, 128, 17]
# edges [isolates v.s. SNPs]	[0, 214, 625, 134, 182, 7515, 434, 4701, 1531, 5887, 129, 5848, 2205, 3663, 416, 2179, 1324, 2311, 2628, 5302, 690, 1347, 2371, 656]
**MDR subset**	
# nodes of each type	[3574, 10, 27, 8, 9, 64, 14, 7, 105, 55, 22, 49, 18, 5, 9, ,135, 7, 57, 44, 24, 16, 15, 11, 15]
# edges [isolates v.s. SNPs]	[0, 176, 490, 116, 135, 7230, 378, 993, 2111, 1223, 2077, 5483, 1949, 59, 320, 1718, 1291, 2023, 2488, 4825, 439, 1387, 107, 594]

Supplement B

In this supplement, we first provide details for PMI-based embedding initialisation, followed by details of the proposed HGAT-AMR model.

B.1 PMI-based embedding

The parameters required before PMI-based embedding include embedding dimension (denoted *d-in*), cut-off thresholds of counts for most and the least frequent SNPs in all isolates; as words in all sentence (denoted *th-most* and *th-least*, respectively). The corresponding values used in this paper are *d-in*=128, *th-most*=90% of the total number of isolates, *th-least*=3 counts. Given these thresholds, the most and least frequent SNPs are removed from the full dataset. PMI-based embedding method generates embedding for the remaining SNPs. Then, the embedding of isolates is obtained by aggregating the resulting embedding of SNPs with a maximum operator. Finally, the embedding of isolates and SNPs are used to be initialised embedding for HGAT-AMR model in the next step.

B.2 Architecture of HGAT-AMR

The hyperparameters of HGAT-AMR and the details of MTB graphs constructed based on different subsets are listed in table below.

Table S.2 Hyper-parameters of HGAT-AMR

**Hyper-parameter name**	**Value**
# Heads of HGAT module	2
# Layers of HGAT module	2
Drop-out rate	0.1
**The 1st HGAT layer**	
Input embedding dimension	128
Output embedding dimension	64
Attention dimension	50
Gamma	0.1
**The 2nd HGAT layer**	
Input embedding dimension	64
Output embedding dimension	64
Attention dimension	50
Gamma	0.1
**The dense layer before last layer**	
Input dimension	64*4
Output dimension	1

Supplement C

In this supplement, we provide hyperparameter sets in grid search for classical machine learning comparators, followed by pipeline of evaluating conventional machine learning comparator models (as shown in Figure S.1).

Figure S.3 Details of MTB graphs

Model	Parameter sets
SVM	{"rbf", gamma: [1e-3, 1e-4], C: [1, 10, 100, 1000]}<br>{"linear", C: [1,10,100,1000]}
Logistic regression	C: [0.01, 0.1, 1, 10, 100], penalty: [L1, L2]
Random forest	n_estimators: linspace(200, 2000,10)<br>max_features: [auto, sqrt]<br>max_depth: linspace(10,110,11)<br>min_samples_split: [2,5,10]<br>min_samples_leaf: [1,2,4]<br>bootstrap: [True, False]

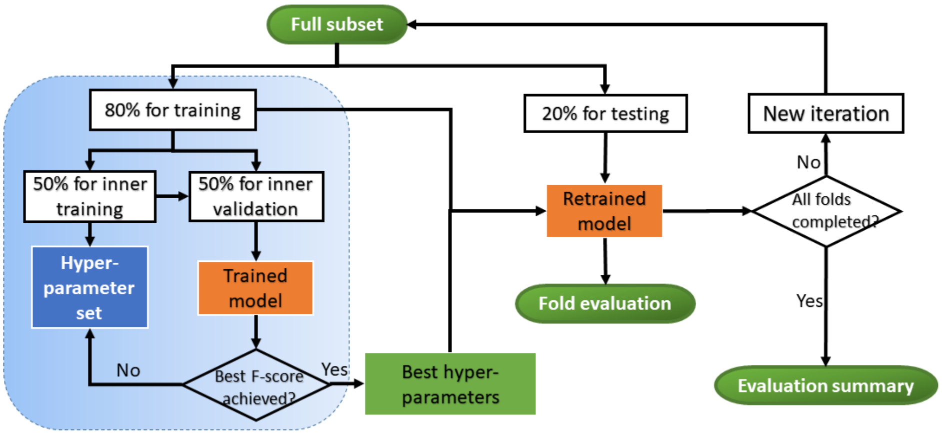

Figure. S.1 Pipeline of evaluating classical machine learning models

Supplement D

In this supplement, we provide details for training the HGAT-AMR, followed by a pipeline of inductive training.

While training HGAT-AMR, an optimiser of Adam was applied, the learning rate was set to 0.005, weight decay was set to 5e-5, the maximum epochs was set to 400. An early stop criterion was applied, when the validation accuracy keeps to increase for 15 epochs, the model stops to be optimised and the resulting model is save as the best model. While evaluation, the threshold on predicted probability is set to 0.5.

Figure S.2 shows pipeline for training HGAT-AMR, where two modes of inductive learning are illustrated in the left yellow box.

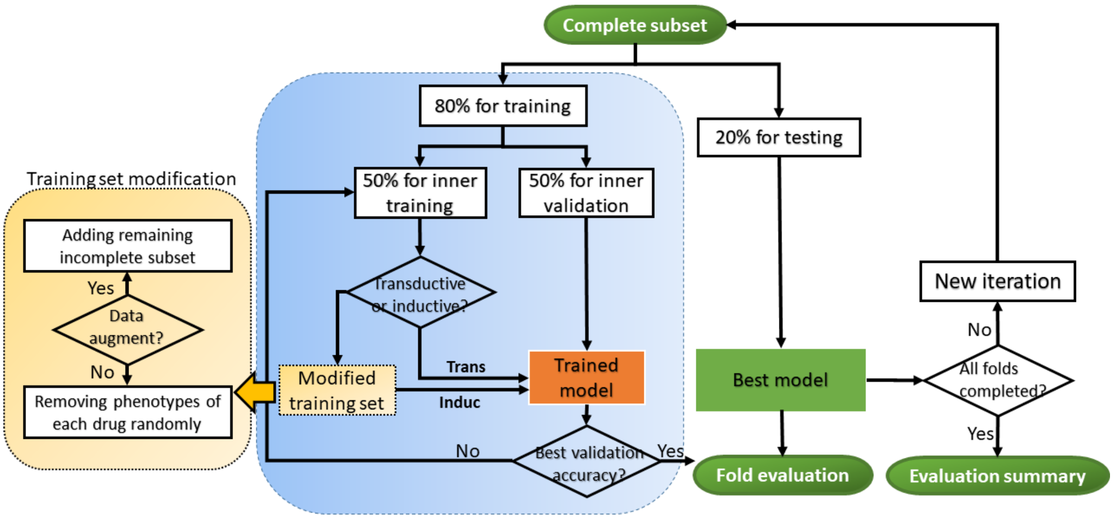

Figure. S.2 Pipeline of evaluating HGAT-AMR model

Supplement E

In this supplement, we provide averaged gene-level attentions scores obtained by HGAT-AMR for each of the four first-line drugs (Figure S.3). Also, we provide another example to demonstrate the node-level attention (Figure S.4).

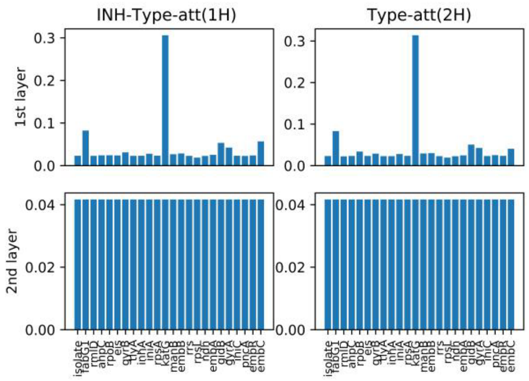

(a) INH

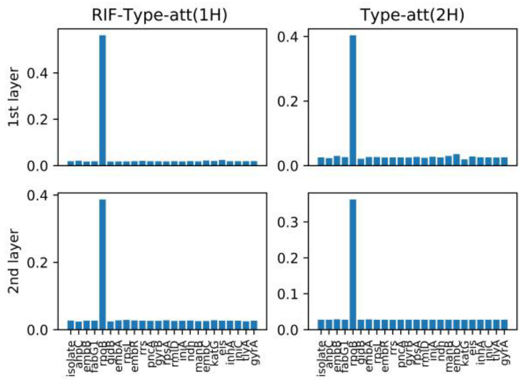

(b)RIF

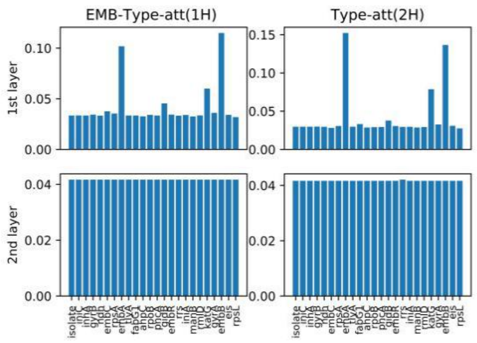

(c) EMB

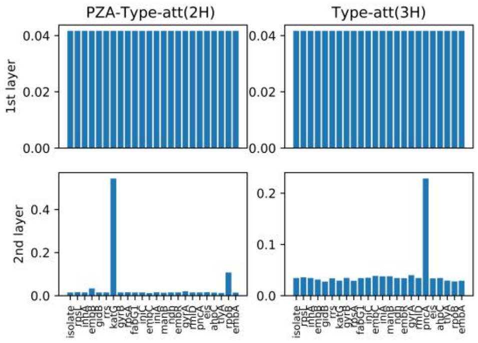

(d) PZA

Figure S.3. Averaged type-level attention scores for (a)INH, (b)RIF, (c)EMB and (d)PZA

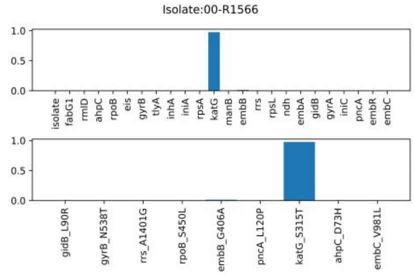

(a)INH (1L1H)

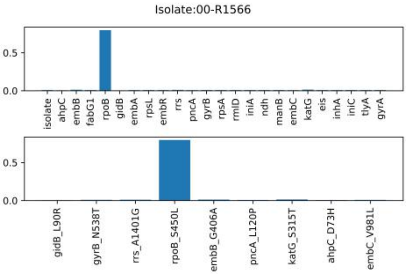

(b)RIF (2L1H)

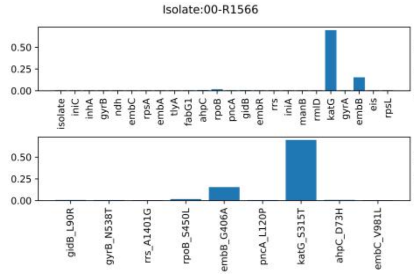

(c)EMB (1L2H)

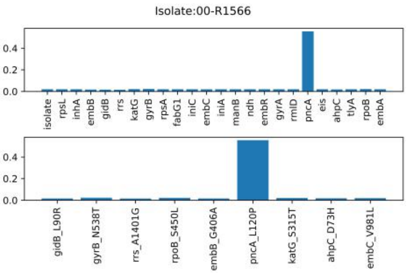

(d)PZA (2L3H)

Figure S.4. the isolate “00-R1566”: averaged isolate-level attention scores for (a) INH; (b) RIF; (c) EMB and (d) PZA.

Supplement F

In this supplement, the performance metrics of three models, LR, SVM and HGAT for predicting INH, PZA, EMB and RIF are listed in table S4-7

Table S4 Performance results for INH with different label-masking and class imbalance ratio

Mask	Imb	LR-Sens	SVM-Sens	HGAT-Sens	LR-Precision	SVM-Precision	HGAT-precision	LR-AUPRC	SVM-AUPRC	HGAT-AUPRC
0.2	1/6	97.43	97.61	92.85	95.87	90.64	94.48	96.67	95.84	95.17
0.2	1/3	88.91	89.8	90.85	95.8	95.43	95.63	95.49	93.68	93.17
0.2	1/2	93.55	96.08	94.43	96.83	96.38	97.97	98.15	97.88	97.97
0.2	1/1.5	91.57	94.19	96.37	96.52	96.83	97.78	98.33	98.19	99.15
0.2	1	92.61	93.13	93.9	95.44	94.86	96.19	98.46	97.78	98.59
0.4	1/6	96.04	95.96	89.93	95.72	92.39	92.49	95.74	95.36	93.03
0.4	1/3	88.73	88.7	91.97	95.58	95.18	95.74	95.3	94.11	97.5
0.4	1/2	93.35	95.57	96.68	96.8	96.28	97.62	97.94	97.6	98.67
0.4	1/1.5	91.34	93.3	95.6	96.32	96.53	97.58	98.11	97.9	98.75
0.4	1	92.24	92.9	93.9	95.08	94.79	95.77	98.15	97.68	98.74
0.6	1/6	93.48	94.37	85.34	95.57	91.36	95.79	95.13	94.92	92.61
0.6	1/3	87.42	85.84	93.57	95.27	94.83	97.1	95	94.13	97.73
0.6	1/2	92.81	94.78	93.03	96.54	95.79	97.26	97.78	97.27	98.23
0.6	1/1.5	91.33	91.99	94.27	96.29	96	97.47	98.34	97.65	98.34
0.6	1	91.23	91.87	95.34	94.83	94.25	96.98	98	97.65	99.31
0.8	1/6	88.52	88.61	96.78	95.46	92.27	96.8	93.56	94.01	96.67
0.8	1/3	85.44	83.89	92.3	95.22	94.44	95.93	94.6	94.04	92.81
0.8	1/2	91.14	91.83	95.92	95.94	94.29	96.77	97.4	96.8	98.64
0.8	1/1.5	89.63	90.29	93.89	95.48	94.8	97.04	97.41	97.19	98.39
0.8	1	89.62	90.77	95.22	94.07	93.49	97.05	97.93	97.5	99.31
0.9	1/6	80.74	78.96	87.69	94.82	92.67	93.95	92.83	93.06	91.82
0.9	1/3	82.98	81.82	92	94.7	93.4	95.52	94.29	94.29	96.62
0.9	1/2	89.35	90.83	93.4	95.48	93.48	97.64	96.91	96.88	98.23
0.9	1/1.5	86.58	87.3	94.97	94.74	93.47	97.12	96.92	97	98.34
0.9	1	87.77	86.66	95.16	93.33	92.16	96.09	97.58	97.23	99.17
0.95	1/6	68.13	60.78	88.23	92.26	91.05	85.63	91.3	92.28	84.42
0.95	1/3	75.86	70.58	95.35	94.22	93.12	98.13	93.61	93.9	98.1
0.95	1/2	85.01	83.95	95.83	94.76	92.36	96.09	96.05	96.52	97.96
0.95	1/1.5	84	83.57	94.51	93.49	92.09	97.03	96.2	96.61	98.6
0.95	1	85.19	84.96	95.36	92.14	91.03	96.92	97.03	96.99	98.4

Table S5 Performance results for PZA with different label-masking and class imbalance ratio

		LR-Sens	SVM-Sens	HGAT-Sens	LR-Precision	SVM-Precision	HGAT-precision	LR-AUPRC	SVM-AUPRC	HGAT-AUPRC
0.2	1/6	74.09	80.14	75.91	81.11	75.54	74.34	76.78	74.64	64.64
0.2	1/3	82.78	88.76	81.13	83.88	81.49	81.03	84.99	81.98	78.06
0.2	1/2	85.69	87.21	84.3	85.71	85.28	83.16	84.24	81.93	84.63
0.2	1/1.5	86.4	87.06	85.58	88.13	88.54	83.13	91.17	89.42	84.27
0.2	1	86.06	83.71	83.79	85.51	85.15	85.54	88.39	87.07	91.58
0.4	1/6	73.78	78.75	78.81	80.28	75.75	75.21	75.3	72.59	74.96
0.4	1/3	83.19	87.73	89.48	83.54	80.99	80.48	83.55	81.03	78.73
0.4	1/2	85.49	87.58	84.04	85.39	85.04	84.6	84.24	81.67	86.11
0.4	1/1.5	86.06	85.58	89.84	87.58	87.93	86.82	90.24	88.84	86.87
0.4	1	85.2	83.76	86.75	85.22	84.9	84.92	88.04	86.29	85.84
0.6	1/6	70.87	80.17	90.51	79.13	74.53	74.3	71.96	69.73	69.37
0.6	1/3	80.69	86.41	87.33	82.7	80.9	83.14	80.87	78.77	82.2
0.6	1/2	84.42	85.71	87.48	84.94	84.64	86.51	83.98	81.68	87.72
0.6	1/1.5	85.42	85.39	87.45	87.22	87.16	87.41	89.49	88	86.25
0.6	1	85.8	81.79	84.76	85.23	84.06	85.58	86.23	85.9	90
0.8	1/6	64.58	70.84	82.41	78.47	75.02	74.13	67.52	64.75	72.04
0.8	1/3	77.48	83.14	85.54	81.85	80.26	80.53	77.19	75.96	76.09
0.8	1/2	82.14	83.09	88.47	84	83.83	86.43	82.48	81.53	86.02
0.8	1/1.5	83.98	82.53	89.61	86.1	85.77	86.9	87.89	86.82	89.87
0.8	1	84.64	80.31	89.93	84.28	83.12	87.49	84.34	84.05	91.81
0.9	1/6	57.73	60.06	85.71	77.13	75.2	73.33	63.36	60.02	71.08
0.9	1/3	73.49	76.88	87.31	81.15	79.22	81.05	74.6	72.77	76.63
0.9	1/2	77.81	75.51	89.86	83.08	82.34	86.34	81.47	79.68	88.06
0.9	1/1.5	80.21	75.49	86.61	84.65	83.91	85.78	86.68	84.91	88.56
0.9	1	82.01	71.56	90.02	83.07	80.42	86.25	83.67	83.71	90.92
0.95	1/6	48.42	50.79	85.64	76.95	75.04	75.85	58.66	55.11	73.67
0.95	1/3	64.96	64.96	92	79.79	78.69	79.71	71.31	70.21	75.22
0.95	1/2	69.03	65.82	90.57	81.04	80.49	83.55	79.79	78.35	81.99
0.95	1/1.5	71.73	62.93	90.1	82.7	81.08	85.13	85.42	83.6	85.14
0.95	1	73.32	63.13	88.34	80.11	77.52	87.55	82.89	82.37	91.33

Table S6 Performance results for EMB with different label-masking and class imbalance ratio

		LR-Sens	SVM-Sens	HGAT-Sens	LR-Precision	SVM-Precision	HGAT-precision	LR-AUPRC	SVM-AUPRC	HGAT-AUPRC
0.2	1/6	86.84	85.79	76.44	73.74	72.45	75.01	64.47	65.93	62.4
0.2	1/3	88.7	88.63	83.55	84.32	83.54	83.39	83.06	82.28	78.35
0.2	1/2	86.5	84.66	88.4	85.03	85.51	88.08	84.38	84.57	88.28
0.2	1/1.5	88.62	87.5	88.49	87.43	87.62	87.33	90.79	88.24	90.73
0.2	1	82.37	77.85	90.79	80.67	80.03	85.86	79.58	75.74	94.61
0.4	1/6	86.21	85.43	65.82	73.3	72.88	72.22	67.43	64.85	60.61
0.4	1/3	87.57	89.19	90.46	83.93	83.26	82.7	82.34	82.27	76.91
0.4	1/2	85.89	85.02	88.95	84.57	85.2	85.41	83.57	83.5	88.25
0.4	1/1.5	89.19	87.44	90.33	87.24	87.25	86	89.83	87.71	87.94
0.4	1	80.59	78.26	93.13	80.58	80.11	86.51	78.83	75.68	95.43
0.6	1/6	83.19	84.43	77.48	73.55	72.26	70.81	65.48	63.43	59.11
0.6	1/3	85.8	86.96	89.05	83.37	82.6	78.94	81.51	80.53	77.1
0.6	1/2	84.68	84.54	90.88	83.77	84.13	88.76	81.6	80.78	88.6
0.6	1/1.5	88.73	87.73	89.62	86.81	86.8	87.86	88.66	86.57	90.48
0.6	1	79.51	79.34	92.19	80.72	79.6	86.95	78.55	75.01	96.77
0.8	1/6	78.12	80.76	75.97	72.86	71.8	70.08	62.03	59.75	54.89
0.8	1/3	80.98	84.14	86.39	82.64	81.81	81.39	79.81	78.46	80.28
0.8	1/2	82.59	83.11	89.13	83.01	83.18	86.6	79.31	78.21	85.02
0.8	1/1.5	87.33	88.26	91.5	86.1	86.11	86.98	86.55	84.7	90.71
0.8	1	74.1	75.21	93.24	80.33	79.42	87.26	76.51	74.53	96.61
0.9	1/6	71.37	74.31	86.12	71.74	71.24	74.02	57.53	55.63	66.14
0.9	1/3	73.75	77.68	91.47	81.56	80.85	78.98	77.73	75.6	74.51
0.9	1/2	78.74	78.77	90.44	82.36	82.13	85.88	78.16	76.64	86.5
0.9	1/1.5	84.71	84.79	93.8	85.24	85.19	88.94	85.17	83.9	90.93
0.9	1	64.96	59.44	90.96	78.89	78.79	86.18	73.48	72.5	91.2
0.95	1/6	63.34	62.55	86.51	72.61	70.53	73.12	54.83	50.88	65.45
0.95	1/3	66.09	65.43	92.29	80.56	79.31	82.84	75.81	72.84	81.52
0.95	1/2	71.67	68.55	89.04	81	80.57	82.61	76.32	75.23	78.49
0.95	1/1.5	79.34	73.46	93.21	83.56	82.32	88.34	83.72	82.87	93.08
0.95	1	57.42	53.56	92.27	77.88	77.11	85.31	72.8	70.79	94.98

Table S7 Performance results for RIF with different label-masking and class imbalance ratio

		LR-Sens	SVM-Sens	HGAT-Sens	LR-Precision	SVM-Precision	HGAT-precision	LR-AUPRC	SVM-AUPRC	HGAT-AUPRC
0.2	1/6	95.44	97.36	90.31	91.74	88.32	97.06	95.29	94.9	95.47
0.2	1/3	94.37	95.58	93.87	96.56	93.05	93.55	98.16	97.34	96.35
0.2	1/2	96.14	96.94	95.75	96.1	95.04	97.44	97.98	98.12	97.87
0.2	1/1.5	93.76	94.07	95.6	96	96.04	97.42	97.91	97.69	98.89
0.2	1	95.56	95.64	95.28	97.44	97.33	96.44	99.43	99.34	99.45
0.4	1/6	95.78	98.11	94.24	91.7	86.96	95.32	94.64	94.52	95.14
0.4	1/3	94.39	98.67	93.29	96.67	93.31	95.7	97.94	97.15	97.7
0.4	1/2	95.64	96.57	97.65	96	94.69	96.7	97.78	97.87	98.84
0.4	1/1.5	93.59	93.59	95.47	95.86	95.72	96.85	97.78	97.78	98.49
0.4	1	95.4	95.4	93.06	97.15	97.01	95.81	99.39	99.39	99.24
0.6	1/6	92.89	96.17	90.5	91.5	87.62	93.81	93.7	94.03	93.79
0.6	1/3	93.65	93	94.47	96.53	93.77	96.33	97.78	97.2	96.36
0.6	1/2	94.31	96.1	93.43	95.62	94.32	97.3	97.59	97.67	98.61
0.6	1/1.5	93.28	93.59	92.94	95.66	95.12	95.18	97.62	97.39	98.55
0.6	1	94.26	94.54	93.42	96.56	96.39	95.8	99.34	99.24	99.23
0.8	1/6	87.25	91.86	97.26	92.1	89.44	92.48	92.25	93.43	98.67
0.8	1/3	92.61	90.78	90.42	95.92	94.07	92.63	97.4	97.31	94.37
0.8	1/2	92.5	93.59	94	94.67	93.92	96.27	97.35	97.47	98.36
0.8	1/1.5	92.05	91.89	95.63	95.09	94.37	97.27	97.53	97.35	99.32
0.8	1	92.24	93.42	95.21	95.5	95.26	95.79	99.15	99.06	99.22
0.9	1/6	77.72	85.72	89.86	91.88	89.49	91.15	90.61	92.11	91.33
0.9	1/3	88.41	89.73	96.02	95.37	93.23	94.49	97.16	97.03	97.72
0.9	1/2	89.64	90.04	93.47	93.88	93.27	96.55	96.85	97.38	97.66
0.9	1/1.5	90.5	88.6	93.26	94.27	93.71	95.86	97.24	97.22	98.61
0.9	1	90.49	89.66	94.49	94.28	93.66	95.9	98.86	98.77	99.18
0.95	1/6	57.42	60.28	96.21	91.64	89.67	90.19	88.9	89.72	95.95
0.95	1/3	83.69	76.87	95.58	94.25	92.07	94.11	96.73	96.52	97.24
0.95	1/2	86.86	83.64	94.67	92.6	91.8	95.9	96	96.93	98.53
0.95	1/1.5	86.52	84.63	94.1	93.39	92.27	96	96.7	96.77	98.69
0.95	1	88.6	86.26	94.2	93.16	92.37	94.95	98.54	98.6	98.96
